# Right Atrial Perforation Leading to Cardiac Tamponade Following Veno-Venous Extracorporeal Membrane Oxygenation Cannulation

**DOI:** 10.7759/cureus.13157

**Published:** 2021-02-05

**Authors:** Jasmine Sandhu, Ryan K Dean, David Landsberg

**Affiliations:** 1 Internal Medicine, State University of New York Upstate Medical University, Syracuse, USA; 2 Internal Medicine/Critical Care, State University of New York Upstate Medical University, Syracuse, USA; 3 Internal Medicine/Critical Care, Crouse Hospital, Syracuse, USA

**Keywords:** tamponade, ecmo, cannulation, vv ecmo, ecmo complication

## Abstract

Extracorporeal membrane oxygenation (ECMO), as a supportive modality for cardiopulmonary failure, is increasing in its use due to improved advances in technology and experience lending to availability and ease of implementation. Complications with ECMO are quite common, and with increasing use, an increase in complications are a natural result. These complications can be from the underlying disease process or from the ECMO process itself, including cannula insertion. One such complication includes perforation of surrounding structures at site of insertion. We will present a case of right atrial perforation after single lumen cannula insertion, which led to development of cardiac tamponade and subsequently cardiac arrest. In addition to cannula design, lack of wire rigidity can play a role in wire migration and injury to surrounding structures. We emphasize the importance of ultrasound guidance and surveillance with echocardiogram or fluoroscopy during ECMO cannulation, regardless of cannula type, to prevent fatal complications.

## Introduction

There has been a dramatic increase in extracorporeal membrane oxygenation (ECMO) usage over the last decade. What was once a tool used primarily for the pediatric population, has now also become an essential tool in the management of adults with severe cardiopulmonary failure, not amenable to conventional therapy. Despite its frequent use, complications remain quite common, whether related to the underlying disease pathology or as a direct result of the ECMO circuit itself [1]. These include bleeding, which is often exacerbated by heparin infusions, systemic thromboembolism, intracerebral hemorrhage/infarct or other neurologic sequelae, hemolysis, heparin-induced thrombocytopenia, hypertension, and septic complications [2]. In general, veno-venous (V-V) ECMO has shown to have fewer complications than veno-arterial (V-A) ECMO, and those circuits started for pulmonary support have fewer complications than those started for cardiac support. Cannulation related complications occur less than 5%, but with their own share of morbidity. These include vessel perforation, arterial dissections, and incorrect placement location [3].

## Case presentation

A 54-year-old male with a history of chronic obstructive pulmonary disease had reported worsening dyspnea and productive cough for one week. He was found to be hypoxic and then intubated due to ongoing respiratory distress, despite high flow ventilation. A computed tomography (CT) of the chest showed diffuse, patchy ground-glass opacities throughout bilateral lungs. He was started on broad-spectrum antibiotics and placed on airway pressure release ventilation (APRV). He improved from a respiratory perspective and was extubated on hospital day four, however, due to worsening respiratory distress and hypoxia he was reintubated on hospital day nine. Over the next 36 hours, the patient was hypoxemic despite increasing P high settings on APRV and 100% fraction of inspired oxygen (FiO2) and the decision was made to start V-V ECMO. 

**Figure 1:**
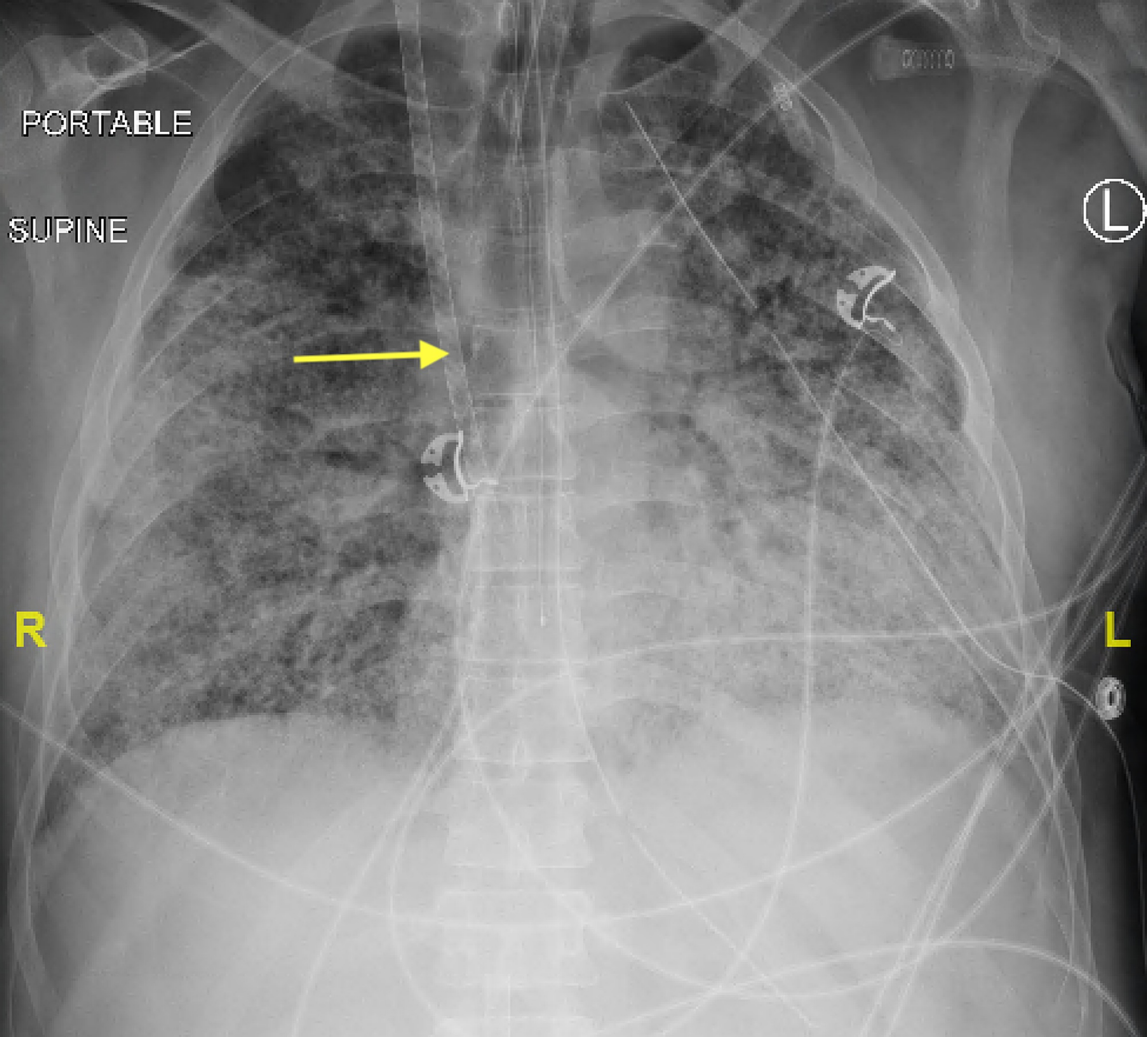
Portable supine anteroposterior radiograph of the chest demonstrate presence of right internal jugular catheter (yellow arrow) terminating in the right atrium. No evidence of pneumothorax. Cardiomediastinal contours are difficult to evaluate due to overlying opacities.

Under ultrasound guidance, a 17-French return cannula (Bio-Medicus, Minneapolis, MN, USA) was percutaneously inserted into the right internal jugular vein (IJ) via Seldinger technique, with tip of cannula at the junction of superior vena cava (SVC) and right atrium. Next, a 21-French Bio-Medicus drainage cannula was placed in the right common femoral vein via Seldinger technique, with the tip in the inferior vena cava (IVC). The patient tolerated cannulation without any complication. A chest radiograph confirmed appropriate placement of IJ catheter (Figure 1).

Shortly after, during placement of the arterial line, the patient went into pulseless electrical activity (PEA) and cardiopulmonary resuscitation (CPR) was initiated. A bedside echocardiogram revealed a large pericardial effusion demonstrating tamponade physiology (Video 1).

**Video 1:** Parasternal long axis view showing large pericardial effusion with blood flow from right atrium into pericardial space.

After an emergent bedside pericardiocentesis, with 60cc of blood drained, the patient remained in PEA. A 17-French cannula was inserted through the left common femoral artery into the aorta and the patient was converted to V-A ECMO. Once the V-A ECMO circuit was started, CPR was ceased, and the patient maintained acceptable hemodynamics. He underwent an emergent median sternotomy, the pericardial sac was incised with cautery, and approximately one liter of dark blood was evacuated from the pericardial sac. There was immediate relief of tamponade and improvement of hemodynamics. On inspection, a clear injury to the right atrium at the junction of the IVC was identified and subsequently repaired. The patient was weaned off ECMO on postoperative day 12 and he underwent tracheostomy on postoperative day 17. The patient was eventually discharged to a vent facility one month later. 

## Discussion

One specific complication that is overlooked during ECMO cannulation is cardiac perforation. The historical rate of hemorrhagic pericardial tamponade obtained from the Extracorporeal Life Support Organization (ELSO) registry was 0.53% from 1985 to 2010 [4]. Interestingly, some studies have suggested that perforation rates may be related to cannula design [4,5]. In V-V ECMO, cannulation can be undertaken utilizing either a bicaval, double lumen single cannula approach or a single lumen two cannula approach. In the single lumen, double cannula circuit, the cannulas are commonly placed, percutaneously via the Seldinger technique, in the right internal jugular vein to the level of the superior vena cava/right atrium and the right femoral vein to the level of the inferior vena cava. Alternatively, bilateral femoral vein cannulas can be placed as well. The two cannula technique has shown to have limitations of gas exchange efficiency due to recirculation at high blood flow rates and also affects patient mobility; hindering early mobilization in these critically ill patients [6]. Thus, the bicaval, dual lumen single cannula was developed to improve on these limitations. The cannula is introduced via the right internal jugular vein, passing through the right atrium and into the inferior vena cava. Proper insertion technique and precise positioning are needed for this cannula type. Serious cannula complication can occur and right heart perforation with resultant tamponade has been well described in the literature, with an incidence of 3-15% [7].

While cardiac perforation may be well described using bicaval cannulas, the incidence is far less common using a single lumen, double cannula circuit. The literature surrounding these perforations are sparse, whether the incidence is truly is uncommon, or perhaps, underreported. Certainly, these specific complications can be dependent on the operator. However, it has also been postulated that the guidewires used in ECMO cannula packages may be more prone to causing perforation. Most of the ECMO packages contain soft, flexible guidewires, as opposed to more stiff guidewires often used in interventional cardiology procedures. The lack of rigidity of these guidewires may result in greater chance of intravascular or intracardiac “looping” that directs the dilators and cannulas toward the cardiac wall, and increasing risk of perforation [8]. Some studies have reported no significant changes in flow through mispositioned cannula therefore we should not solely rely on flow characteristics to identify proper placement [4]. The current ELSO guidelines suggest cardiac evaluations via point of care ultrasound at pre-ECMO, during ECMO initiation and post-ECMO phases. These evaluations can determine cardiac function prior to ECMO, detect and prevent complications intra-procedure such as malposition, kinking and excessive guidewire use, and can detect post-procedure pathologies such as perforations and tamponade [9]. 

Prompt recognition of cannula insertion complications is critical. The use of intraoperative ultrasound has been suggested superior to ultrasound scan for ECMO cannula placement [10,11]. There has been a report of prompt identification of cardiac tamponade due to right ventricular perforation because of intraoperative transesophageal echocardiogram (TEE) use [12]. Implementation of frequent echocardiograms, during and after cannula placement, can potentially identify these minor position changes that can lead to detrimental complications. Additionally, some experts have suggested the use of fluoroscopy, if available, during cannulation to decrease risk [8,12]. Transferring a patient to a fluoroscopy suite may not be feasible when the patient is unstable and minimizing radiation exposure should be considered. 

## Conclusions

Although ECMO cannulation technique can vary depending on the operator and cannula design, frequent cardiac evaluations with point-of-care ultrasound is an easily accessible, non-invasive method to quickly identify cannulation complications. Therefore, ultrasound, whether point-of-care or TEE, should be considered routine evaluations during cannulation to avoid serious complications such as this. While specific cannula types have been associated with increased risk of injury, the overall rate of this complication is quite low. Despite this, it is imperative for clinicians to be aware of this complication regardless of the cannula type that is used, so that it may be recognized and treated promptly due to the large morbidity and mortality with which it is associated. 
